# The Impact of Dietary Habits and Nutrition Knowledge on Harmful Alcohol Use and Nicotine Dependence Among Medical Students: A Single-Center, Cross-Sectional Study

**DOI:** 10.3390/nu17111788

**Published:** 2025-05-24

**Authors:** Aureliusz Andrzej Kosendiak, Bartosz Bogusz Adamczak, Zofia Kuźnik, Szymon Makles, Weronika Hariasz

**Affiliations:** 1 College of Health Studies, University of Lower Silesia, 53-611 Wroclaw, Poland; aureliusz.kosendiak@dsw.edu.pl; 2 Medical Faculty, Wroclaw Medical University, 50-345 Wroclaw, Poland; 3 Medical Faculty, University of Opole, 45-040 Opole, Poland

**Keywords:** nutrition knowledge, alcohol, nicotine, substance use, medical students

## Abstract

**Background:** Harmful alcohol use and nicotine dependence are major public health concerns. One group particularly at risk may be medical students, who might resort to substance use as a coping mechanism for stress. Various factors may influence these behaviors, either positively or negatively—among them, dietary knowledge and eating habits. **Methods**: In this study, we used the KOMPAN questionnaire to assess dietary habits and nutrition knowledge, the AUDIT questionnaire to evaluate problematic alcohol consumption, and the Fagerström Test to assess nicotine dependence. A total of 2801 medical students participated in this study, including 2374 alcohol users and 379 smokers. **Results**: Smoking students demonstrated significantly lower dietary quality (*p* < 0.0001) and nutrition knowledge (*p* = 0.0004). Among alcohol users, lower levels of problematic alcohol consumption were observed in individuals with better dietary quality (*p* = 0.0006) and higher nutrition knowledge (*p* < 0.0001). **Conclusions**: Future research should explore additional factors contributing to the clustering of unhealthy behaviors and the underlying causes of alcohol consumption and poor dietary habits among healthcare professionals.

## 1. Introduction

Substance abuse remains one of the most prevalent and detrimental coping mechanisms employed in response to psychological distress [1]. Within the medical profession, where individuals are regularly exposed to high levels of stress, irregular working hours, sleep deprivation, and frequent encounters with traumatic events [2], the incidence of substance misuse is notably higher. A study conducted in the United States involving 7288 physicians from various medical specialties reported that 12.9% of male participants and 21.4% of female participants met the diagnostic criteria for alcohol abuse or dependence [3]. Alarmingly, these patterns of behavior are already observed among medical students [4], who, despite being at an earlier stage in their professional development, experience considerable academic pressure, intense competition, and frequent emotional exhaustion [5]. An American study involving over 4000 medical students found that more than 30% of participants met the diagnostic criteria for alcohol abuse or dependence [6]. A 2022 study conducted on a similar cohort of medical students from Wrocław, Poland, found that one-fourth of the participants currently smoke or have smoked cigarettes in the past [7]. This finding is consistent with studies of the general adult Polish population, in which approximately 25% of adults report being active or occasional smokers [8]. Early adoption of maladaptive coping strategies such as substance use during medical education may predispose these individuals to continued harmful behaviors throughout their professional careers [9]. This pattern may be understood within the framework of the Stress and Coping Model, which explains that individuals assess stressful situations through a process of cognitive appraisal and subsequently adopt coping strategies to manage the perceived stress. In the case of medical students and professionals, substance use may represent a specific form of emotion-focused coping, employed to reduce feelings of anxiety, emotional exhaustion, or psychological burden resulting from academic or occupational stressors [10].

In Poland, alcohol and nicotine are the most commonly misused substances, largely due to their legal status, social acceptability, and ease of accessibility [11]. Both substances pose substantial threats to public health [12]. Alcohol misuse is associated with a wide range of adverse outcomes, including liver disease, cardiovascular problems, mental health disorders, and increased risk of accidents and injuries [13,14]. Similarly, nicotine dependence significantly contributes to the burden of non-communicable diseases, notably cardiovascular disease, chronic respiratory conditions, and multiple forms of cancer [15]. Given these serious health implications, understanding the factors that contribute to substance use is of critical importance, particularly within this vulnerable population of medical students.

Another potentially harmful coping mechanism is the adoption of unhealthy dietary patterns [16]. Emerging evidence suggests a complex relationship between diet and smoking, as well as alcohol consumption [17,18]. Individuals who adhere to healthier dietary habits and possess higher levels of dietary knowledge (DK) are more likely to engage in a range of positive health behaviors and exhibit lower rates of substance misuse [19]. Conversely, poor dietary patterns and insufficient understanding of nutrition may correlate with heightened stress, emotional dysregulation, and engagement in risky behaviors, including harmful alcohol consumption and smoking [20]. While DK is an important determinant of dietary behavior, it is essential to analyze these variables independently, as existing data indicate that there is a significant yet only weak positive correlation between higher levels of DK and higher dietary intake of fruit and vegetables [21].

Medical students, given the biomedical focus of their curriculum, are expected to have a relatively high level of nutrition literacy [22]. However, despite their knowledge, it is plausible that they may not consistently adhere to healthy dietary guidelines, possibly due to the cumulative stressors inherent in their training. A 2017 study examining first-year medical students revealed that 59% to 93% accurately identified the recommended daily fruit servings, while 61% to 84% correctly recognized the vegetable intake recommendations. Despite this knowledge, only 40% to 46% of students achieved the recommended fruit consumption levels, and merely 12% to 19% met the guidelines for vegetable intake [19].

As future healthcare providers and role models for patients, medical students’ lifestyle behaviors warrant close examination. Their current health behaviors not only impact their own well-being but may also influence their future clinical practices and patient counseling strategies [23]. Understanding the interplay between dietary habits, DK, and substance use among medical students is thus essential for informing the development of effective, targeted health promotion interventions [24].

The objective of this study is to investigate the impact of dietary habits and nutrition knowledge on harmful alcohol use and nicotine dependence among medical students. Specifically, this study aims to determine whether healthier dietary patterns and higher levels of DK are associated with lower rates of alcohol and nicotine use. We hypothesize that medical students with healthier dietary patterns and higher levels of nutrition knowledge will report lower levels of harmful alcohol use and nicotine dependence. By identifying potential relationships, this research seeks to provide a scientific foundation for the development of preventive strategies and tailored health education programs within medical curricula, thereby supporting the promotion of healthier lifestyles among future healthcare professionals.

## 2. Materials and Methods

### 2.1. Study Design and Participants

This study was conducted as a cross-sectional survey among students at Wroclaw Medical University. Its primary objective was to explore the relationship between dietary habits, nutrition knowledge, and the risk of harmful alcohol use and nicotine dependence. Data were collected over two years, from 2022 to 2023. All students enrolled in mandatory physical education classes were invited to participate in an anonymous online survey, which was distributed through dedicated links. To ensure broad participation, invitations were sent to all students who met the inclusion criteria. Physical education classes are mandatory for all students, regardless of their physical condition. For students with disabilities, these classes take a more theoretical form or involve physical activity adapted to what is feasible for the individual. A total of 6411 students took part in this study. The survey links were distributed to specific student groups during physical education classes. The university’s physical education instructors supervised the distribution process to ensure that individual students did not complete the questionnaire more than once. Recruitment was carried out twice each academic year—at the beginning of each semester—with the survey remaining open until the start of the following term.

**Figure 1:**
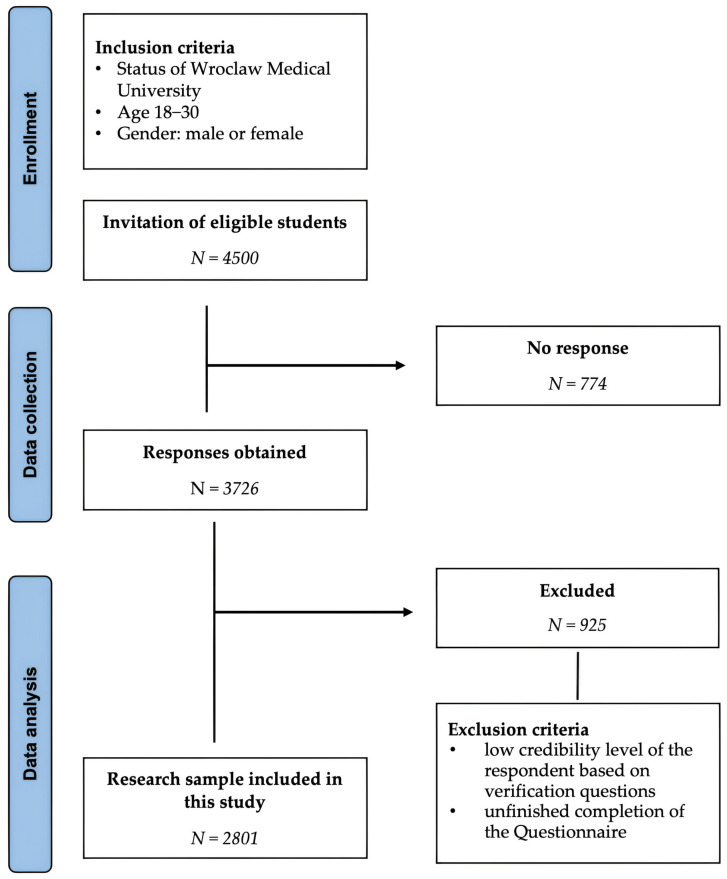
Study selection process.

Only students who fully completed the questionnaire were included in the final analysis; incomplete responses were excluded. A detailed description of the recruitment process, along with inclusion and exclusion criteria, is presented in Figure 1.

Participation in this study was entirely voluntary. Students were informed about the purpose of the research and asked to provide informed consent before completing the questionnaire. This study followed established ethical guidelines to ensure data confidentiality and participant rights.

This study was conducted in accordance with the Declaration of Helsinki and was approved by the Ethics Committee of the Wroclaw Medical University (No. KB-251/2020, approval date: 17 March 2020).

### 2.2. KomPAN

The assessment of dietary habits and nutrition-related beliefs was carried out using the KomPAN questionnaire [25], developed by the Polish Academy of Sciences. This tool is widely recognized for its reliability in evaluating individual dietary patterns and nutritional knowledge.

The first part of the questionnaire addresses the frequency of consumption across various food groups through 33 items—of which 24 are used to calculate two indices: the Non-Healthy Diet Index (NHD) and the Pro-Healthy Diet Index (PHD). The NHD reflects the frequency of intake of 14 food categories considered potentially harmful to health, while the PHD covers 10 groups considered beneficial. Based on their responses, participants receive a score that reflects their dietary tendency as low (0–33%), medium (34–66%), or high (67–100%).

To further analyze dietary quality, the Diet Quality Index (DQI) was calculated by subtracting the nHDI-14 percentage score from the pHDI-10 score. This produces a value ranging from −100 to 100, allowing for insight into whether an individual’s overall dietary pattern is more health-promoting or health-risking. Based on their responses, participants receive a score that reflects their dietary tendency as low (−100–−26), medium (−25–25), or high (25–100).

The second part of the KomPAN survey is a single-choice test assessing nutrition knowledge. It consists of 25 statements, with response options being “True”, “False”, or “I’m not sure”. One point is awarded for each correct answer, while incorrect or uncertain responses receive zero points. The total score reflects the participant’s level of dietary knowledge (DK). Based on their responses, participants receive a score that reflects their dietary tendency as insufficient (0–8), sufficient (9–16), or good (17–25).

To ensure response reliability, three sets of validation questions were included throughout the survey. Responses that did not meet these reliability checks were excluded from the analysis. The KomPAN tool has undergone thorough validation and is regarded as a reliable instrument for research into dietary behavior and nutritional awareness [26,27]. Cronbach’s alpha for this questionnaire ranged from 0.70 to 0.90.

### 2.3. The AUDIT

Alcohol use was assessed using the Alcohol Use Disorders Identification Test (AUDIT), developed by the World Health Organization [28]. The tool consists of 10 items divided into three categories: hazardous alcohol use (questions 1–3), dependence symptoms (questions 4–6), and harmful alcohol use (questions 7–10).

Based on their total score, respondents were classified as follows: a score of 8–15 suggests hazardous drinking; 16–19 indicates harmful drinking; and a score of 20 or more may reflect probable alcohol dependence. The AUDIT questionnaire has been extensively validated and is widely used in clinical and research settings [29].

### 2.4. The Fagerström Test

Nicotine dependence was measured using the Fagerström Test for Nicotine Dependence, an internationally recognized tool comprising six items [30,31,32]. Each item is scored between 0 and 3 points, depending on the response. The sum of these scores determines the level of nicotine addiction: scores of 0–4 suggest no or minimal dependence (indicating habitual rather than pharmacological smoking); 5–8 points imply moderate dependence, where the individual may struggle to abstain in stressful situations or under social pressure; and 9–11 points indicate high nicotine dependence, often accompanied by smoking-related health issues. The Fagerström test has been validated and is considered a reliable measure of physical addiction to nicotine [33,34].

### 2.5. Statistical Analysis

All collected data were compiled using Microsoft Excel (version 16.77, Redmond, WA, USA) and analyzed with Statistica 13 software (StatSoft, Kraków, Poland). The Shapiro–Wilk test was used to assess the normality of data distribution and revealed that the data did not follow a normal distribution.

Descriptive statistics were calculated, including frequency counts for categorical variables and medians with interquartile ranges (IQRs) for continuous variables. For comparisons involving two independent groups, the Mann–Whitney U test was used. When comparing a continuous variable across three or more independent groups, the Kruskal–Wallis test was applied. Additionally, the k-means clustering method was applied, utilizing Euclidean distance as the measure of distance between observations. The optimal number of clusters was determined through v-fold cross-validation, ensuring the best fit for the data structure. The following variables were used in the model: Diet Quality Index (DQI), Dietary Knowledge (DK), and AUDIT scores. All statistical tests were conducted with a significance level set at *p* < 0.05.

## 3. Results

### 3.1. Characteristics of Study Participants

Table 1 presents the demographic and baseline characteristics of the study participants. The majority were women (77%). Approximately 85% of participants reported alcohol consumption and were non-smokers. Most participants scored low in both PHD (82.1%) and NHD (97.1%) categories, with only a few individuals reaching high levels in these dimensions. The overall dietary quality, as measured by the DQI, indicated that the majority (87.6%) belonged to medium DQI, reflecting average dietary quality, while only a few were classified in low DQI, indicating below-average diet quality. More than half of the participants demonstrated sufficient nutritional knowledge, while only 13.6% had insufficient knowledge.

### 3.2. Differences Between Drinking Alcohol and Smoking Status on Study Variables

Table 2 shows the associations between alcohol consumption and smoking status with the study variables. For alcohol consumption, the only statistically significant difference was found in DK, which was higher among those who reported drinking. In contrast, smokers showed significantly lower levels of PHD, DQI, and DK and higher NHD scores compared to non-smokers.

### 3.3. Differences Between Study Variable Levels and AUDIT Domain Scores

Table 3 explores the relationships between levels of dietary habits and nutrition knowledge with the domains of the AUDIT among participants who consumed alcohol.

For the PHD and NHD variables, the High group was excluded from the statistical analysis due to the small sample size. For the DQI and DK variables the Low group was excluded for the same reason.

Due to limited group sizes, only two groups were compared for PHD, NHD, and DQI variables, while all three groups were analyzed for DK. Across all domains and variables, a consistent trend emerged: individuals with healthier dietary habits (i.e., higher PHD and DQI scores and lower NHD scores) and higher DK tended to have lower AUDIT scores. The only exception was the HazAU domain in relation to PHD, where this association was not statistically significant.

### 3.4. Differences Between Study Variable Levels and Fagerstrom Test for Nicotine Dependence Total Scores

Table 4 presents the associations between dietary habits, dietary knowledge, and total scores on the Fagerström Test for Nicotine Dependence (FTND) among smokers. For the PHD and NHD variables, the High group was excluded from the statistical analysis due to the small sample size. For the DQI and DK variables, the Low group was excluded for the same reason.

Due to small group sizes, only two groups were compared for PHD, NHD, and DQI, while three groups were compared for DK. Participants with lower NHD levels had significantly lower FTND scores. Additionally, a higher level of DK was associated with lower nicotine dependence. Differences in FTND scores related to PHD and DQI were not statistically significant.

### 3.5. Cluster Analysis—AUDIT Total Score

Table 5 summarizes the cluster analysis based on DQI, DK, and total AUDIT scores. Clusters 1 through 3 were characterized by low AUDIT scores but varied in terms of DQI and DK levels. Cluster 5, which had the highest AUDIT score, also showed the lowest levels of DQI and DK. Notably, Cluster 3, despite a similarly low DQI score, had a much lower AUDIT score compared to Cluster 5, while having nearly three times higher DK scores. Interestingly, Cluster 4, despite higher DQI and DK levels, had an AUDIT score more than three times higher than that of Cluster 3.

### 3.6. Linear Regression Analysis

Table 6 presents the results of separate linear regression analyses for the AUDIT and FTND scores. In both cases, no significant associations were found with the PHD and DQI variables. However, in both cases, a positive relationship was observed with NHD and a negative one with DK.

## 4. Discussion

This study investigates the impact of dietary habits and nutrition knowledge on harmful alcohol use and nicotine dependence among students at Wroclaw Medical University. The cohort, predominantly female, reflects the gender distribution typical of medical universities [35]. Participants were primarily from Wroclaw, an urban center with over 100,000 residents, though some commuted from rural areas.

Nearly 85% of the study participants were classified as alcohol consumers. This percentage is higher than that reported in the 2019 Eurostat statistics, which indicated that 75% of the general population in Poland consumes alcohol [36], as well as findings from another Polish study showing that 80% of Polish students engage in alcohol consumption [37]. Moreover, a 2020 study involving Polish medical students found that 94% of respondents reported consuming alcohol within the past year [38]. In contrast, the proportion of smokers was markedly lower, with only 13.5% of the cohort reporting nicotine use. This finding is lower than the smoking prevalence reported in the general Polish population, where 18.4% are smokers [39]. According to a comparative study of Italian and Polish dental students, the prevalence of smoking was lower among Polish students (28%) than among their Italian counterparts (42%), with both rates notably higher than the prevalence observed in our study cohort [40]. Our findings are also more favorable compared to the aforementioned 2022 study on a cohort of medical students from Wrocław, in which approximately one-fourth of the participants were active smokers [7].

The Pro-Healthy Diet (PHD) variable was assessed based on the frequency of consumption of foods such as fish, whole grains, vegetables, etc. Only 20% of students were classified as having a medium or high level of adherence to a PHD. Conversely, the Non-Healthy Diet (NHD) variable, based on the intake of foods such as white bread, processed foods, sweets, alcoholic beverages, etc., indicated that 97.1% of participants were classified as having a low level of unhealthy dietary patterns. These findings are reflected in the Diet Quality Index (DQI), where 87.6% of participants were categorized as having an average diet quality. Regarding DK, only 13.6% of students demonstrated insufficient DK, whereas the majority exhibited sufficient (58.2%) or good (28.2%) levels of DK. These results align with the expectation that medical students, because of the content of their curriculum, possess a moderate level of nutrition-related knowledge, which appears to be reflected in their overall average diet quality.

Research on the topic of medical students’ dietary habits remains limited. A 2021 Spanish study conducted among university students found that 17.4% of participants maintained a healthy diet, with poor compliance with the recommendations, highlighting especially the low levels of “fruit” and “vegetables” that they consumed, as well as high levels of “cold meats and cuts” and “sweets”, which is consistent with the DQI scores of our study participants, indicating irregular adherence to PHD [20]. A 2015 Italian study involving medical students reported that over 70% of participants had very limited knowledge regarding healthy eating, a significantly worse outcome compared to the 13.6% of students with insufficient DK in our study [41].

In our study, smokers demonstrated significantly lower levels of PHD, DQI, and DK, as well as higher NHD scores compared to non-smokers. These findings suggest that being a smoker is strongly associated with unhealthy dietary behaviors. This association may reflect a broader tendency to adopt multiple harmful coping mechanisms, with nicotine use being linked to poor dietary choices.

In Poland, various strategies have been implemented over the years to reduce smoking prevalence. These measures included nationwide campaigns (such as the Great Smoke-Out campaign), a ban on the sale of tobacco to minors and via vending machines, a ban on tobacco advertising across all media, the introduction of textual health warnings on cigarette packaging, and the free provision of smoking cessation treatment [42]. These efforts have contributed to increased public awareness and the development of a social stigma surrounding smoking. Additionally, nearly half of the adult population supports further strengthening of anti-tobacco policies, with over 40% advocating for a total ban on tobacco sales [43]. Consequently, it is plausible that medical students who engage in smoking, despite the associated social stigma and well-documented health risks, may also be more prone to other detrimental lifestyle behaviors, including poor dietary choices.

This relationship is also reflected in our findings from Table 4, where participants with medium NHD scores exhibited higher levels of nicotine dependence compared to those with low NHD scores. Moreover, lower levels of DK were associated with higher nicotine dependence scores. Among smokers, greater nicotine dependence was associated with higher NHD scores and lower DK scores. However, the associations with PHD and DQI were not statistically significant, suggesting that while higher nicotine dependence may be linked to poorer dietary choices, lower nicotine dependence is not necessarily associated with the implementation of better dietary habits and an overall healthier diet. Previous research has demonstrated that the diet quality of heavy smokers is significantly poorer than that of individuals who have never smoked, regardless of socioeconomic, lifestyle, or biological confounding factors. Heavy smokers have also been shown to exhibit lower adherence to any dietary recommendations [44]. A 2024 study involving over 3000 participants found that smokers exhibited a significant 90% increase in the frequency of consuming frozen meals and pizzas over the past 30 days compared to non-smokers [45]. Another study, which included 80,296 men and women, revealed that smokers were more likely to skip meals, go without food for more than three hours, add salt and sugar to their meals, overeat, and struggle to leave food on their plates compared to non-smokers. Additionally, smoking was associated with a higher frequency of consuming fried foods per week when compared to non-smokers [46].

Regarding alcohol consumption, the only statistically significant difference observed between drinkers and non-drinkers in our study was in DK, which was higher among individuals who reported alcohol use. No significant differences were found between drinkers and non-drinkers concerning PHD, NHD, or DQI, which may be attributed to the much higher prevalence of alcohol consumption compared to smoking within the study cohort, as in Polish society, alcohol use is considerably more socially accepted.

The substantial cultural differences in perceptions of smoking versus drinking may be reflected in our findings. This could account for the absence of statistically significant associations between alcohol consumption and PHD, NHD, and DQI observed in our study. The difference between social perception of smoking versus drinking may be illustrated by the limited implementation of the WHO’s “best buys” strategies when it comes to alcohol consumption [47]. In contrast to Poland, the Baltic countries successfully introduced effective measures, including substantial increases in alcohol excise taxes, restrictions on sales hours, and comprehensive advertising bans, leading to reductions in alcohol consumption and alcohol-related mortality rates [48]. Meanwhile, Poland systematically weakened its regulatory framework by reducing excise taxes on spirits, liberalizing beer advertising, and expanding alcohol availability, contributing to increased consumption and higher alcohol-related mortality. As a result, frequent and occasional alcohol use in Poland has become widely normalized, often incorporated into meals, celebrations, national holidays, post-work relaxation, and even as a common form of gifting [49].

According to a 2022 M. Caliendo and J. Hennecke study, individuals who generally engage in health-conscious behaviors, such as maintaining a healthy diet, may simultaneously partake in alcohol consumption, particularly when such behavior is socially integrated and perceived as moderate or responsible [50]. As suggested by existing research, moderate alcohol use is often associated with positive social, economic, and even some health outcomes, aligning with the broader pattern of proactive lifestyle choices typically seen in individuals with a stronger internal, self-driven responsibility belief [51]. Furthermore, it has been proposed that these individuals may overestimate their ability to manage and prevent the negative consequences of drinking, leading them to underestimate its associated risks [52]. This mechanism may explain why adherence to healthy dietary practices can coexist with alcohol consumption among health-conscious individuals observed in our study.

However, our findings indicate that individuals exhibiting healthier dietary patterns, reflected by higher PHD and DQI scores, lower NHD scores, and greater DK, were less likely to demonstrate symptoms of hazardous or harmful alcohol use and alcohol dependence. This suggests that heavy and problematic drinkers are more prone to engage in other detrimental health behaviors, such as maintaining an unhealthy diet and possessing inadequate DK, which is a well-studied phenomenon [53]. Scientific research indicates that increased consumption of alcoholic beverages is correlated with a deterioration in dietary quality [54]. Higher levels of alcohol intake are commonly associated with reduced consumption of fruits and an elevated intake of calories derived from alcohol itself, as well as from foods high in unhealthy fats and added sugars [55]. Additionally, individuals consuming alcohol in high quantities demonstrate a significantly higher caloric intake compared to those with low consumption, while also exhibiting a lower fiber intake relative to low-quantity drinkers [56]. Such a pattern aligns with the broader concept of health behavior clustering, where positive or negative behaviors tend to co-occur [57]. Consequently, individuals who invest effort into maintaining healthier nutrition and acquiring DK may be more inclined toward overall health-conscious lifestyles, thereby reducing their harmful alcohol consumption.

Additionally, our findings indicate that among drinking medical students, greater DK was associated with a substantially stronger protective effect against alcohol dependence symptoms (DepSymp), while exhibiting a smaller influence on hazardous alcohol use (HazAU) behaviors. This finding suggests that DK may be more effective in enhancing self-regulatory behaviors and health awareness relevant to recognizing DepSymp, whereas HazAU appears to be more strongly driven by external social and environmental influences, such as peer pressure, and therefore less responsive to health-related knowledge [58].

Additionally, our cluster analysis revealed several notable findings. The comparison between Cluster 3 and Cluster 5 suggests that while poor dietary habits may be associated with heavy alcohol consumption, higher levels of DK could serve as a protective factor against excessive drinking, potentially through enhanced awareness of the health risks linked to alcohol use. In contrast, the findings from Cluster 4 challenge the assumption that superior DK invariably is associated with reduced alcohol consumption. This suggests that while some individuals may benefit from the protective effects of DK in reducing alcohol abuse, others with adequate DK may nonetheless engage in harmful drinking behaviors. Such behaviors are likely driven by external social, psychological, or cultural influences, such as peer group norms that encourage excessive drinking during social gatherings [59], or the use of alcohol as a coping mechanism for academic stress [60].

This underlines the urgent need for multifactorial public health interventions that address behavioral, psychological, and cultural determinants of a healthy lifestyle among university students. Beyond individual educational efforts, there is a critical need for more structured governmental initiatives and the legislative implementation of the WHO’s “Best Buys” recommendations regarding alcohol use. Greater emphasis should be placed on nationwide campaigns aimed at de-normalizing casual drinking, similar to the successful strategies employed in anti-smoking efforts.

Although our sample predominantly comprised female participants—reflecting the current gender distribution within medical student populations—it is noteworthy to acknowledge that potential gender differences may have influenced the results. Existing research indicates that dietary habits, substance use, and associated health risks may differ substantially by gender. For instance, men are more likely to consume larger quantities of animal protein, while women more frequently report consumption of sweets and high-fat, salty snacks [61]. Similarly, gender-specific patterns have been observed in substance use behaviors; men continue to report higher levels of alcohol consumption overall. However, recent trends suggest a narrowing gender gap, with alcohol use rising among women and declining among men [62]. Furthermore, gender differences have been identified in the psychosocial mechanisms underlying smoking behavior, with women more likely to cite emotional and stress-related factors as barriers to cessation, in contrast to men, who are more influenced by environmental cues [63].

Our findings illustrate that while higher nutrition knowledge is associated with healthier behaviors, it does not alone prevent the adoption of harmful practices such as excessive alcohol consumption and smoking. Medical universities, given their critical role in shaping future healthcare providers, must take an active role in promoting comprehensive lifestyle education that integrates practical behavioral change strategies with traditional curricular content. Future interventions should prioritize high-risk groups, such as smokers, individuals with unhealthy dietary habits, and those prone to substance abuse. Tailored programs aimed at eliminating non-healthy dietary habits are critical, especially given the evidence that smoking and poor diet often coexist. These programs should not only focus on raising awareness but also on changing behaviors through practical solutions such as personalized nutrition plans, cooking classes, and targeted campaigns that promote healthier food choices. Additionally, special attention must be given to addressing the psychological and environmental factors that contribute to these behaviors. The integration of mobile apps for dietary tracking, individual consultations with nutritionists, and regular workshops can empower individuals to implement dietary knowledge effectively and improve overall diet quality. Beyond individual educational efforts, there is a critical need for more structured governmental initiatives and the legislative implementation of the WHO’s “Best Buys” recommendations regarding alcohol use. The implementation of policies such as a sugar tax and reduced taxes on healthy foods can create an environment that incentivizes healthier choices. These initiatives should be complemented by nationwide public health campaigns that focus on de-normalizing unhealthy behaviors like excessive alcohol consumption and smoking, mirroring the success of anti-smoking initiatives. It is also important for programs aimed at individuals affected by substance abuse to incorporate strategies for improving dietary habits. By supporting substance users in making healthier food choices, these programs can address both immediate and long-term health risks, providing a more holistic approach to substance abuse recovery. Future research should prioritize the identification of additional components contributing to the clustering of unhealthy behaviors, as well as the underlying causes of alcohol consumption and poor dietary habits among future healthcare professionals.

Several specific interventions should be implemented to ensure that this study’s findings contribute to practical institutional actions. Integrating targeted lifestyle education into the medical curriculum is crucial; such education should include evidence-based content on nutrition, substance use risks, and stress management, alongside practical workshops on meal planning and healthy cooking facilitated by dietitians and mental health professionals.

Additionally, establishing support programs that combine smoking cessation with nutritional counseling could more effectively address the co-occurrence of these unhealthy behaviors. Promoting the use of mobile health applications for self-monitoring diet and substance use may further enhance students’ self-regulation and awareness.

Our findings suggest that avoiding poor dietary choices—and not just increasing healthy ones—may be particularly important in reducing risky behaviors. Likewise, while better nutrition knowledge is generally helpful, it does not always translate into healthier actions for everyone. This highlights the need for integrated campaigns that address both diet and substance use together. A coordinated approach is more likely to reach students who might not respond to single-issue interventions, and it reflects the real-world overlap between these behaviors.

Fostering interdisciplinary collaboration between university health services, counseling centers, and public health agencies is recommended to provide comprehensive support for students at risk. Moreover, universities should actively engage in national health promotion campaigns and endorse the implementation of the WHO’s “Best Buys” policies on tobacco and alcohol control to help de-normalize harmful behaviors through coordinated public awareness efforts.

These combined efforts can help medical students build healthier habits, not only for their own well-being but also in preparation for their future roles as health professionals.

While this study offers meaningful insights into the relationships between dietary habits, DK, and substance use among medical students, several limitations must be acknowledged. The sample consisted exclusively of students from Wroclaw Medical University, representing a relatively homogenous and academically specialized population, which restricts the generalizability of the findings to wider, more diverse populations. Additionally, the use of an online, self-administered questionnaire introduces the potential for self-reporting bias and misinterpretation of questions, despite efforts to ensure clarity in survey design. Moreover, participants’ responses may have been affected by social desirability bias, especially regarding behaviors such as alcohol use and smoking. This bias, reflecting a preference to align with perceived social norms, may have contributed to the underreporting of such behaviors and, consequently, to an underestimation of their actual prevalence in the sample. As the data rely solely on subjective declarations, the absence of objective health measures, such as body composition analysis, limits the precision of the health behavior assessments. In future research, the integration of both objective physical health metrics and the recruitment of a more diverse, representative sample will be crucial to enhance the external validity and broader applicability of the findings.

## 5. Conclusions

This study aimed to investigate the impact of dietary habits and nutrition knowledge on harmful alcohol use and nicotine dependence among medical students at Wroclaw Medical University. The present study found that poor dietary habits and lower DK are strongly associated with nicotine dependence and, to a lesser extent, harmful alcohol use among medical students. Smokers exhibited notably poorer adherence to a PHD, lower overall diet quality, and inferior DK compared to non-smokers. These findings are consistent with previous research indicating that smoking behaviors cluster with multiple other unhealthy lifestyle patterns.

While alcohol consumption was highly prevalent within the cohort, its association with dietary habits differed from that of smoking. General alcohol use did not have a strong relationship with poor diet quality, a finding that may reflect the broader normalization and social acceptance of alcohol in Polish society. These findings align with previous studies suggesting that while moderate alcohol consumption may coexist with otherwise health-conscious behaviors, heavy drinking often clusters with other forms of health risk.

Importantly, although students generally exhibited sufficient levels of DK—a result superior to findings reported among other European medical students—this knowledge alone was not always protective against unhealthy substance use. Cluster analysis revealed that even individuals with high DK sometimes engaged in harmful alcohol consumption. A possible explanation for this finding, backed by existing research, is that harmful drinking behaviors are frequently shaped by external social, psychological, and cultural factors, such as peer group norms that encourage excessive alcohol consumption during social gatherings and the utilization of alcohol as a coping mechanism for academic-related stress.

Higher nutrition knowledge alone does not prevent harmful behaviors such as excessive alcohol consumption and smoking, highlighting the need for comprehensive lifestyle education in medical universities. Future interventions should focus on high-risk groups, including smokers and those with unhealthy dietary habits, through tailored programs that incorporate personalized nutrition plans, cooking classes, and behavioral change strategies. Governmental policies, such as a sugar tax and reduced taxes on healthy foods, should be implemented to incentivize healthier choices, alongside public health campaigns to de-normalize excessive alcohol consumption and smoking. Programs targeting substance abuse should also integrate dietary interventions to promote healthier food choices as part of recovery. Future research should explore additional factors contributing to the clustering of unhealthy behaviors and the underlying causes of alcohol consumption and poor dietary habits among healthcare professionals.

## Figures and Tables

**Table 1:** Characteristics of study participants.

Variables	Total *N* = 2801 [IQR] (%)
**Age mean**	19.8 [19.0–20.0]
**Gender**	
Male	643 (23.0)
Female	2158 (77.0)
**Place of Residence**	
Rural area	690 (24.6)
City < 20,000 *	296 (10.6)
City 20,000–100,000 *	593 (21.2)
City 100,000+ *	1222 (43.6)
**BMI**	
Underweight	113 (4.0)
Normal weight	2352 (84.0)
Overweight and Obese	336 (12.0)
**Drinking Status**	
Drinker	2374 (84.8)
Non-drinker	427 (15.2)
**Smoking Status**	
Smoker	379 (13.5)
Non-smoker	2422 (86.5)
**Pro-Healthy Diet**	
Low	2299 (82.1)
Medium	492 (17.6)
High	10 (0.4)
**Non-Healthy Diet**	
Low	2721 (97.1)
Medium	75 (2.7)
High	5 (0.2)
**Diet Quality Index**	
Low	5 (0.2)
Medium	2454 (87.6)
High	342 (12.2)
**Dietary Knowledge**	
Insufficient	380 (13.6)
Sufficient	1631 (58.2)
Good	790 (28.2)

Note: *N*—the number of observations; *—number of inhabitants.

**Table 2:** Differences between drinking alcohol and smoking status on study variables.

	Mean	Median	IQR	Mean	Median	IQR	*p*-Value	Z
	**Drinkers, *N* = 2374**			**Non-Drinkers, *N* = 427**			**D vs. N-D**	
**Pro-Healthy Diet**	22.95	21.7	14.7–29.5	23.76	21.8	14.1–31	0.65	0.45
**Non-Healthy Diet**	13.81	12.2	8.3–17.2	13.34	11.9	7.5–17.5	0.24	1.18
**Diet Quality Index**	9.15	7.6	0.2–17.0	10.41	7.9	−0.1–19.9	0.27	1.10
**Dietary Knowledge**	13.73	14.0	11.0–17.0	12.41	14.0	9.0–17.0	**0.002**	**3.18**
	**Smokers, *N* = 379**			**Non-Smokers, *N* = 2422**			**S vs. N-S**	
**Pro-Healthy Diet**	21.94	19.6	12.9–28.1	23.25	22.0	14.8–30.0	**0.002**	**3.08**
**Non-Healthy Diet**	15.55	13.3	9.0–19.8	13.45	12.0	8.1–16.9	**0.0009**	**3.33**
**Diet Quality Index**	6.39	5.0	−1.7–13.5	9.80	8.2	0.3–18.2	**<0.0001**	**4.99**
**Dietary Knowledge**	12.72	13.0	10.0–16.0	13.65	14.0	11.0–17.0	**0.0004**	**3.52**

Note: *N*—the number of observations; D vs. N-D—drinkers vs. non-drinkers; S vs. N-S—smokers vs. non-smokers.

**Table 3:** Differences between study variables levels and AUDIT domains scores.

	Mean	Median	IQR	Mean	Median	IQR	Mean	Median	IQR	*p*-Value	Z/H*
	**Pro-Healthy Diet**										
	**Low, *N* = 1967**			**Medium, *N* = 399**			**High, *N* = 8**			**L vs. M**	
**HazAU**	3.73	4.0	2.0–5.0	3.55	3.0	2.0–5.0	5.75	4.5	3.5–8.0	0.22	1.22
**DepSymp**	1.19	0.0	0.0–2.0	1.00	0.0	0.0–1.0	3.75	2.5	0.5–6.0	**0.04**	**2.10**
**HarmAU**	1.45	0.0	0.0–2.0	1.22	0.0	0.0–2.0	3.63	2.5	0.0–6.0	**0.01**	**2.54**
**AUDIT Total**	6.36	5.0	2.0–9.0	5.77	4.0	2.0–7.0	13.13	9.0	6.5–18.0	**0.02**	**2.27**
	**Non-Healthy Diet**										
	**Low, *N* = 2303**			**Medium, *N* = 67**			**High, *N* = 4**			**L vs. M**	
**HazAU**	3.66	3.0	2.0–5.0	4.93	4.0	3.0–6.0	7.75	8.0	5.5–10.0	**0.0001**	**3.88**
**DepSymp**	1.12	0.0	0.0–1.0	2.25	1.0	0.0–4.0	6.00	6.0	1.5–10.5	**0.0001**	**3.88**
**HarmAU**	1.37	0.0	0.0–2.0	2.64	1.0	0.0–4.0	6.00	6.0	2.0–10.0	**0.0008**	**3.36**
**AUDIT Total**	6.16	5.0	2.0–8.0	9.82	8.0	4.0–15.0	19.75	17.5	9.0–30.5	**<0.0001**	**4.10**
	**Diet Quality Index**										
	**Low, *N* = 5**			**Medium, *N* = 2097**			**High, *N* = 272**			**M vs. H**	
**HazAU**	3.20	3.0	3.0–4.0	3.74	4.0	2.0–5.0	3.41	3.0	2.0–4.0	**0.03**	**2.24**
**DepSymp**	1.20	0.0	0.0–2.0	1.22	0.0	0.0–2.0	0.70	0.0	0.0–1.0	**<0.0001**	**4.45**
**HarmAU**	2.60	4.0	0.0–4.0	1.47	0.0	0.0–2.0	0.99	0.0	0.0–1.0	**0.001**	**3.19**
**AUDIT Total**	7.00	9.0	3.0–10.0	6.44	5.0	2.0–9.0	5.10	4.0	2.0–6.5	**0.0006**	**3.45**
	**Dietary Knowledge**										
	**Insufficient, *N* = 281**			**Sufficient, *N* = 1421**			**Good, *N* = 672**			**In vs. S vs. G**	
**HazAU**	4.14	4.0	2.0–6.0	3.70	3.0	2.0–5.0	3.54	3.0	2.0–5.0	**0.008**	**9.75**
**DepSymp**	2.24	1.0	0.0–4.0	1.09	0.0	0.0–1.0	0.87	0.0	0.0–1.0	**<0.0001**	**65.46**
**HarmAU**	2.49	1.0	0.0–5.0	1.33	0.0	0.0–2.0	1.15	0.0	0.0–2.0	**<0.0001**	**41.48**
**AUDIT Total**	8.87	7.0	3.0–14.0	6.12	5.0	2.0–8.0	5.55	4.0	2.0–7.0	**<0.0001**	**36.91**

Note: *N*—the number of observations; HazAU—hazardous alcohol use; DepSymp—dependence symptoms; HarmAU—harmful alcohol use; L vs. M—low vs. medium; M vs. H—medium vs. high; In vs. S vs. G—Insufficient vs. Sufficient vs. Good; H* is given for DK, while Z is given for PHD, NHD, and DQI.

**Table 4:** Differences between study variables levels and Fagerstrom Test for Nicotine Dependence total scores.

	Mean	Median	IQR	Mean	Median	IQR	Mean	Median	IQR	*p*-Value	Z
	**Pro-Healthy Diet**										
	**Low, *N* = 324**			**Medium, *N* = 52**			**High, *N* = 3**			**L vs. M**	
**FTND Score**	2.67	2.0	0.0–4.5	3.00	3.5	0.0–5.0	7.33	8.0	6.0–8.0	0.14	1.49
	**Non-Healthy Diet**										
	**Low, *N* = 356**			**Medium, *N* = 20**			**High, *N* = 3**			**L vs. M**	
**FTND Score**	2.68	2.0	0.0–5.0	3.35	4.0	1.0–6.0	7.33	8.0	6.0–8.0	**<0.0001**	**4.02**
	**Diet Quality Index**										
	**Low, *N* = 1**			**Medium, *N* = 342**			**High, *N* = 36**			**M vs. H**	
**FTND Score**	2.00	2.0	2.0–2.0	2.75	2.0	0.0–5.0	2.81	3.0	0.0–5.0	0.11	1.59
	**Dietary Knowledge**										
	**Insufficient, *N* = 67**			**Sufficient, *N* = 229**			**Good, *N* = 83**			**In vs. S vs. G**	
**FTND Score**	3.42	4.0	1.0–5.0	2.69	2.0	0.0–5.0	2.39	2.0	0.0–4.0	**0.002**	**3.15**

Note: *N*—the number of observations; FTND Score—Fagerstrom Test for Nicotine Dependence total score; L vs. M—low vs. medium; M vs. H—medium vs. high; In vs. S vs. G—Insufficient vs. Sufficient vs. Good.

**Table 5:** Cluster analysis—AUDIT total score.

Cluster Number	Diet Quality Index	Dietary Knowledge	AUDIT Total	*N* (%)
**1**	8.46	8.33	4.05	492 (20.7)
**2**	22.75	18.35	4.14	582 (24.5)
**3**	2.78	14.74	4.25	852 (35.9)
**4**	6.02	14.91	14.67	302 (12.7)
**5**	0.87	5.10	16.90	146 (6.2)

Note: *N*—the number of observations.

**Table 6:** Linear regression analysis.

Cluster Number	B Coefficient ± SE	β Coefficient ± SE	*p*-Value
**AUDIT Total Score**			
**Intercept**	6.513 ± 0.402	-	**<0.0001**
**Pro-Healthy Diet**	−0.008 ± 0.010	−0.017 ± 0.021	0.41
**Non-Healthy Diet**	0.146 ± 0.013	0.223 ± 0.020	**<0.0001**
**Diet Quality Index**	0.0	-	-
**Dietary Knowledge**	−0.150 ± 0.023	−0.14 ± 0.021	**<0.0001**
**FTND Score**			
**Intercept**	0.382 ± 0.088	-	**<0.0001**
**Pro-Healthy Diet**	0.0003 ± 0.002	0.105 ± 0.020	0.88
**Non-Healthy Diet**	0.016 ± 0.003	0.003 ± 0.020	**<0.0001**
**Diet Quality Index**	0.0	-	-
**Dietary Knowledge**	−0.018 ± 0.005	−0.069 ± 0.020	**0.0004**

## Data Availability

The datasets used and analyzed during the current study are available from the corresponding author upon reasonable request. The data are not publicly available due to the inclusion of information that could compromise the privacy of the research participants in accordance with the decision of the Ethics Committee of the Wroclaw Medical University.

## Abbreviations

The following abbreviations are used in this manuscript:

AUDIT	Alcohol Use Disorders Identification Test
BMI	Body Mass Index
DepSymp	Dependence Symptoms
DK	Dietary Knowledge
DQI	Diet Quality Index
FTND	Fagerström Test for Nicotine Dependence
HazAU	Hazardous Alcohol Use
IQR	Interquartile Ranges
NHD	non-Healthy Diet
PHD	pro-Healthy Diet
WHO	World Health Organization
